# Small target tea bud detection based on improved YOLOv5 in complex background

**DOI:** 10.3389/fpls.2024.1393138

**Published:** 2024-06-03

**Authors:** Mengjie Wang, Yang Li, Hewei Meng, Zhiwei Chen, Zhiyong Gui, Yaping Li, Chunwang Dong

**Affiliations:** ^1^ College of Mechanical and Electrical Engineering, Shihezi University, Shihezi, China; ^2^ Key Laboratory of Tea Quality and Safety Control, Ministry of Agriculture and Rural Affairs, Tea Research Institute, Chinese Academy of Agricultural Sciences, Hangzhou, China; ^3^ Tea Research Institute of Shandong Academy of Agricultural Sciences, Jinan, China

**Keywords:** object detection, deep information extraction, lightweight, MPDIoU, YOLOv5, attention mechanism

## Abstract

Tea bud detection is the first step in the precise picking of famous teas. Accurate and fast tea bud detection is crucial for achieving intelligent tea bud picking. However, existing detection methods still exhibit limitations in both detection accuracy and speed due to the intricate background of tea buds and their small size. This study uses YOLOv5 as the initial network and utilizes attention mechanism to obtain more detailed information about tea buds, reducing false detections and missed detections caused by different sizes of tea buds; The addition of Spatial Pyramid Pooling Fast (SPPF) in front of the head to better utilize the attention module’s ability to fuse information; Introducing the lightweight convolutional method Group Shuffle Convolution (GSConv) to ensure model efficiency without compromising accuracy; The Mean-Positional-Distance Intersection over Union (MPDIoU) can effectively accelerate model convergence and reduce the training time of the model. The experimental results demonstrate that our proposed method achieves precision (P), recall rate (R) and mean average precision (mAP) of 93.38%, 89.68%, and 95.73%, respectively. Compared with the baseline network, our proposed model’s P, R, and mAP have been improved by 3.26%, 11.43%, and 7.68%, respectively. Meanwhile, comparative analyses with other deep learning methods using the same dataset underscore the efficacy of our approach in terms of P, R, mAP, and model size. This method can accurately detect the tea bud area and provide theoretical research and technical support for subsequent tea picking.

## 1 Introduction

China is a leading tea-producing country, boasting vast tea cultivation areas and high yields. In 2021, China’s tea gardens covered a total area of 3,217 thousand hectares, yielding 3.16 million tons of tea (Statistics Bureau of the People’s Republic of China, 2021). Despite the extensive cultivation area and output, the picking method for famous teas remains primarily manual, which is both time-consuming and costly. In recent years, the scarcity of tea-picking laborers and the shortened tea-picking season have posed challenges to harvesting famous teas. Consequently, within the current trend of artificial intelligence, the automation and intelligent picking of high-quality tea are becoming imperative. Mechanized tea picking in China currently relies on indiscriminate picking using reciprocating cutting, suitable only for low-quality teas and unable to meet the requirements for the precise bud-by-bud picking of famous tea varieties. Therefore, as artificial intelligence continues to develop, hand-picking methods will likely be replaced by intelligent picking. The intelligent picking of high-quality tea has emerged as a recent research hotspot. Key to this endeavor is the recognition of tea buds, and achieving accurate and rapid tea bud detection will drive the intelligent picking and industrial development of famous teas, holding significant practical significance. This study will thus focus on the precise and rapid detection of tea buds to contribute to the advancement of intelligent tea picking methodologies.

Currently, in the agricultural field, two primary target detection methods are utilized: traditional image segmentation and deep learning (LeCun et al., 2015). Traditional image segmentation methods rely on distinguishing targets such as litchi (Yu et al., 2021), apples (Li et al., 2021), and passion fruits (Tu et al., 2018) from complex backgrounds by leveraging image color, texture, and other features, alongside manually crafted segmentation criteria. For tea bud detection, Zhang et al. (2021) proposed an enhanced watershed algorithm, yielding favorable results in tea bud recognition. Wu et al. (2015) discovered that within the Lab color space, the K-means clustering method exhibited the highest tea bud recognition rate at a shooting distance of 5cm. In these studies, segmentation targets were delineated based on disparities in color and shape. While commendable results have been attained, the intricate tea plantation backgrounds depicted in images present a significant challenge influencing segmentation recognition. Diverse growth environments, lighting conditions, and shooting angles can substantially impede tea bud recognition. Consequently, traditional image segmentation methods often struggle to achieve robust detection outcomes in real-world tea plantation settings.

In recent years, with the development and popularization of deep learning techniques, advanced detection technology has found applications across various agricultural fields. In the detection of small targets such as tea buds, Qian et al. (2020) proposed a semantic segmentation network for tea buds based on TS SegNet, and Hu et al. (2021) proposed a semantic segmentation method based on DP-NET for segmenting and recognizing tea buds in a natural scene, both of which achieved good segmentation results. Although the semantic segmentation method can segment the target significantly, it is complex, slow and difficult to produce a dataset, which is not suitable for large-scale detection models. Therefore, Zhu et al. (2022) explored the Faster R-CNN model and VGG16 feature extraction network to detect the category of tea buds, which significantly improved the model’s detection effect when removing individual buds. Xu et al. (2022) proposed a variable-domain two-level fusion network detection and classification method, which combined the fast detection capability of YOLOv3 and the high-precision classification capability of DenseNet201 to achieve 95.71% accuracy in detecting side buds. Gui et al. (2023) proposed a lightweight tea bud detection model based on YOLOv5_l. Using the Ghost_conv module instead of the original convolution, a floating point operation reduction of 52.402G and a parameter reduction of 22.71M were achieved. Li et al. (2023) proposed a tea bud detection algorithm based on SE-YOLOv5_m. SENet was introduced into the CNN, and the accuracy reached 91.88% by using weights to filter the key features of each convolutional channel.

Ultimately, although numerous scholars have conducted research on tea bud recognition using deep learning, practical application still faces significant challenges such as low detection accuracy, slow processing speed, and high computational costs. These limitations render existing methods unsuitable for deployment on mobile devices, necessitating further research. During the special period of tea bud picking, it is crucial to recognize tea buds quickly and accurately. In this study, we employ YOLOv5 as the foundational network and integrate lightweight and other modules to enhance model accuracy, reduce computational overhead, and enable rapid detection of tea buds amidst complex backgrounds. The specific improvement method is as follows:

1. A Coordinate Attention (CA) mechanism has been integrated after the C3 module in the backbone network to enhance the network’s focus on tea buds.
2. Spatial Pyramid Pooling Fast (SPPF) is applied to the head to deeply extract the semantic information introduced by the enhanced feature extraction network, overcoming the large amount of low-level semantic information in the shallow network that cannot better utilize the information fusion function of the CA_block.
3. A cross-stage partial network (VoV_GSCSP) is used to replace the C3 module in the neck network, ensuring that the model is lightweight without affecting accuracy.
4. Replace the GIoU in the initial network with a new metric of the high-precision boundary regression loss function Mean-Positional-Distance Intersection over Union (MPDIoU), thereby accelerating model convergence and reducing model training time.

In the remainder of the paper, the second section outlines the details of image acquisition, data enhancement, and the overall network structure. The third section presents the test results, while the fourth section concludes the paper.

## 2 Materials and methods

### 2.1 Image acquisition and preprocessing

**Figure 1:**
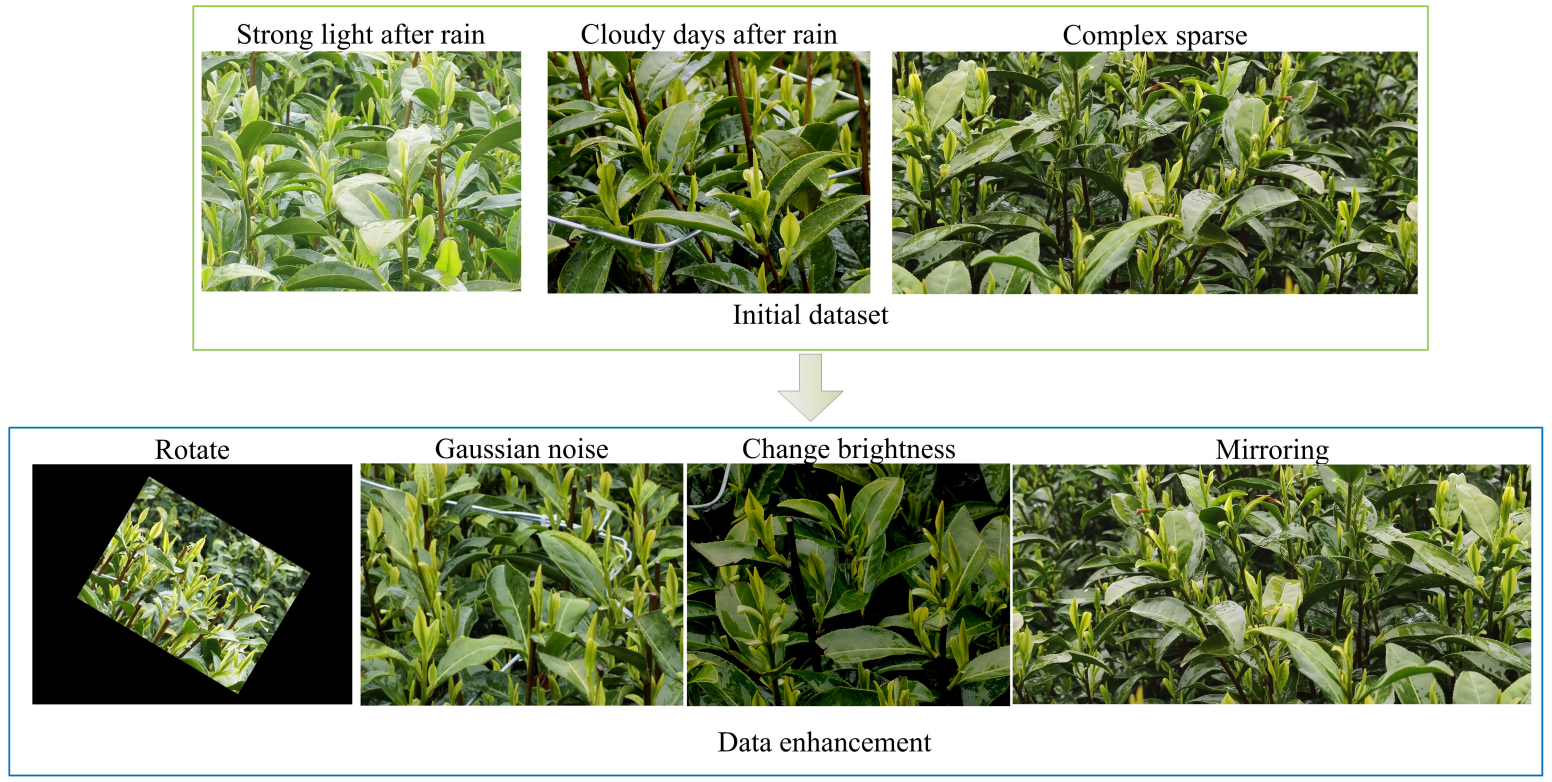
Initial dataset and data augmentation examples.

The tea bud images utilized in this study were obtained from the Shengzhou Tea Base of the Tea Research Institute at the Chinese Academy of Agricultural Sciences (120.825542E, 29.748715N). White tea variety was selected, and the images were captured in March 2022 using Huawei Mate40 and Xiaomi10 smartphones. During the capture, the phones were positioned approximately 0.4 meters away from the tea trees, resulting in a total of 730 images of white tea buds. The images were taken under various conditions, including strong light after rain, cloudy days after rain, and sunny days. The dataset was annotated using Labelimg software. To evaluate the model’s training effectiveness, 73 samples were chosen from the original 730 unprocessed images as the test set. To enhance the generalization capability of the target detection model with limited data, the remaining 657 images underwent augmentation techniques such as mirroring, brightness adjustments, rotation within a range of ±45°, and the addition of Gaussian noise. This augmentation resulted in a total of 1314 tea bud images, which were split into training and validation sets at a ratio of 9:1. Specifically, the training set contained 1182 images, while the validation set contained 132 images. **Figure 1** illustrates an example of the initial dataset and the data augmentation process.

### 2.2 YOLOv5 algorithm

In recent years, the YOLO series has undergone several iterations, with the release of YOLOv5 (Glenn, 2020) in 2020 marking significant advancements in both small target detection accuracy and speed. This model excels in extracting deeper semantic information, rendering it highly adaptable to evolving working scenarios (Gao et al., 2019; Da Costa et al., 2020), resulting in improved recognition precision and robustness (Khosravi et al., 2021). Compared to other YOLO series models, YOLOv5 emerges as the optimal choice for real-time detection of tea gardens in unstructured environments due to its simple network architecture, smaller model size, efficient deployment and operation, and the potential for further speed enhancement through lightweight module integration. In the context of picking famous tea, where detection efficiency directly impacts picking efficiency, selecting YOLOv5 with its superior detection efficiency serves as the initial network for tea bud detection in this study.

In the YOLOv5 network, its size can be adjusted by modifying its width and depth, resulting in four different versions: YOLOv5_s, YOLOv5_m, YOLOv5_l and YOLOv5_x, each with corresponding parameters 7.30 × 10^6^, 2.14 × 10^7^, 4.71 × 10^7^ and 8.78 × 10^7^, respectively. Generally, fewer parameters generally lead to faster computation time but lower precision. Selecting the appropriate variant is key to fully utilizing the power of the YOLOv5 assay. In this study, these four models were trained using the initial dataset without data augmentation, and the results are shown in **Table 1**. YOLOv5_m exhibits the highest detection precision, while YOLOv5_x has the highest recall and average precision. Considering both accuracy and effectiveness in tea bud detection, YOLOv5_m was chosen as the initial network for tea detection, and enhancements were made to its structure for improved performance.

**Table 1:** Test results of the model.

Model	P	R	mAP@0.5(%)	FLOPs(G)	Model size(MB)
YOLOv5_s	80.61	78.61	85.23	**16.477**	**26.95**
YOLOv5_m	**85.20**	78.95	87.78	50.598	80.32
YOLOv5_l	83.39	81.66	88.58	114.559	177.88
YOLOv5_x	82.20	**84.69**	**89.09**	217.795	332.81

Bold values is the optimal value of the comparison results of the four models.

**Figure 2:**
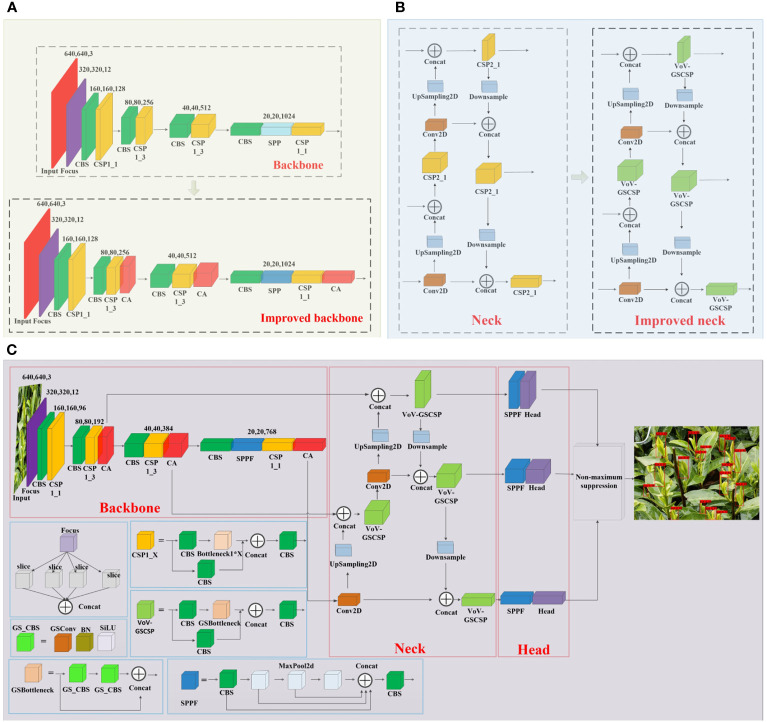
**(A)** The backbone network structure before and after improvement, **(B)** the neck network structure before and after improvement, and **(C)** structure of the improved YOLOv5_m model.

The improved YOLOv5 network structure is shown in **Figure 2C**. In the backbone network, the CA attention mechanism is integrated after all the C3 modules to enhance the model’s focus on target regions and improve its attention towards specific features and contextual details. Additionally, the SPP module is replaced with the SPPF module, enabling the model to effectively capture target information across various scales, thus expanding its perception range and enhancing target detection performance. The backbone module before and after the improvement is shown in **Figure 2A**. In the neck, all the C3 modules are replaced with VoV_GSCSP modules, which aims to fully combine the CA attention mechanism in the backbone with the degree of attention to the target, so that the model can better understand the global information and local details of the target in the image, and ensure the accuracy of the model while making the model lightweight. The neck module before and after the improvement is shown in **Figure 2B**. In the head, to address the challenge of utilizing deep semantic information from the VoV_GSCSP module in the neck layer, an SPPF module is introduced before the head. This module extracts deep semantic features from the neck, further refining the model’s accuracy and performance.

### 2.3 Attention mechanism

**Figure 3:**
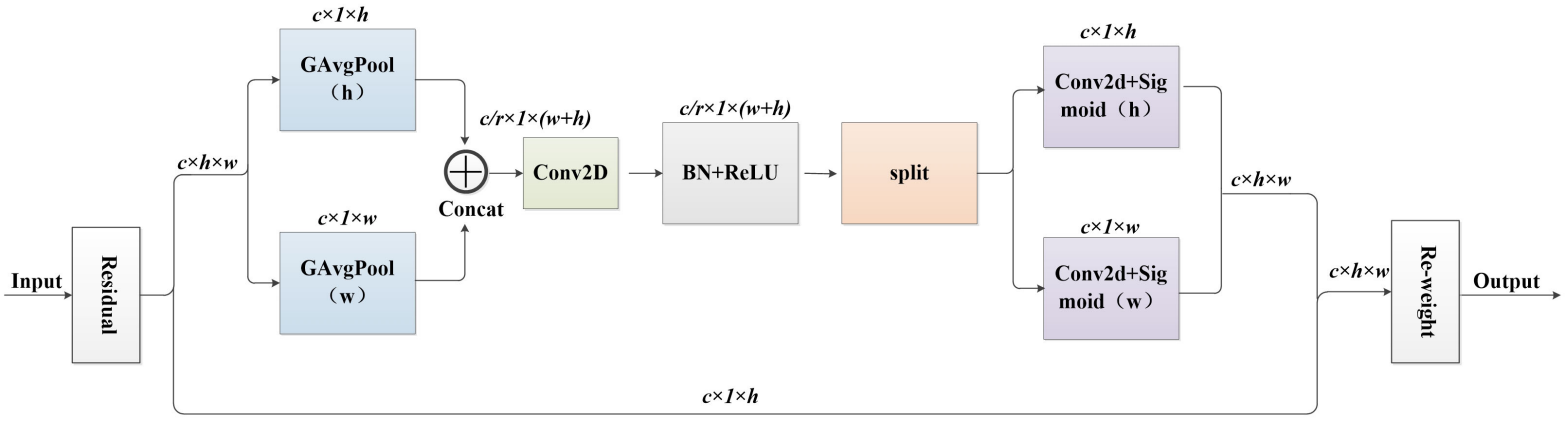
Coordinate attention structure diagram.

To better focus on the overall tea bud, this study introduced the CA_block (Hou et al., 2021) into the C3 module to better extract the deep features of tea buds. Location information is crucial for capturing target structures in visual detection. CA_block is a method of enhancing the interaction and correlation between different channels, which can not only be easily inserted into the core module of a lightweight network, but also capture channel and position information of images, strengthen regions of interest, reduce redundant information, and improve the expression ability of the model to achieve overall attention to tea buds. Its structure is shown in **Figure 3**. It overcomes the problem of the Squeeze-and-Excitation networks (Hu et al., 2018) module only focusing on channel confidence while ignoring spatial information.

CA_block performs global average pooling of feature maps with input size C * H * W from both X and Y directions by obtaining channel and position information, to obtain remote spatial interaction of position information. Mathematical expressions for the feature maps in both directions **Equations 1**, **2**.


(1) $$Z_{c}^{h}\left(h\right)=\frac{1}{W}\sum_{0\leq i\leq W}x_{c}\left(h,i\right)$$

(2) $$\;Z_{c}^{w}\left(w\right)=\frac{1}{H}\sum_{0\leq i\leq H}x_{c}\left(j,w\right)$$

Here, the variable c refers to the channel, and H and W represent the height and width of the input feature map. Specifically, $x_{c}\;$ represents the input x in channel *c*.

Next, concatenate the obtained feature maps, using 1 × 1 convolution module reduces the dimension by 1/r (r is the reduction rate), and after batch standardization and activation function processing, $F_{1}$ is obtained. $F_{1}$ is then activated by the sigmoid function to obtain an intermediate feature map *f* that encodes spatial information in both horizontal and vertical directions. The intermediate feature map *f* can be described as **Equation 3**.


(3) $$f=\delta (F_{1}\left(\left[Z^{h},Z^{w}\right]\right)$$

Where δ denotes the nonlinear activation function.

Subsequently, along the spatial dimension, perform a split operation on *f* to obtain *f^h^* and *f^w^*, using 1 × 1 convolution to dimensionality up operation, and then the feature maps $F_{w}$ and $F_{h}$ are obtained. Then, the attention weights *g^w^* and *g^h^* of the feature maps in height and width are obtained through Sigmoid. It can be mathematically defined as **Equations 4**, **5**.


(4) $$g^{h}=\sigma \left(F_{h}\left(f^{h}\right)\right)\;$$

(5) $$g^{w}=\sigma \left(F_{w}\left(f^{w}\right)\right)$$

Where *σ* denotes the sigmoid function.

Finally, the attention weight feature map for both the h and w directions is obtained by multiplying and weighting the feature map. The calculation method is shown in **Equation 6**.


(6) $$y_{c}\left(i,j\right)=x_{c}\left(i,j\right)\times g_{c}^{h}\left(i\right)\times g_{c}^{w}\left(j\right)$$

### 2.4 Cross-stage partial network

**Figure 4:**
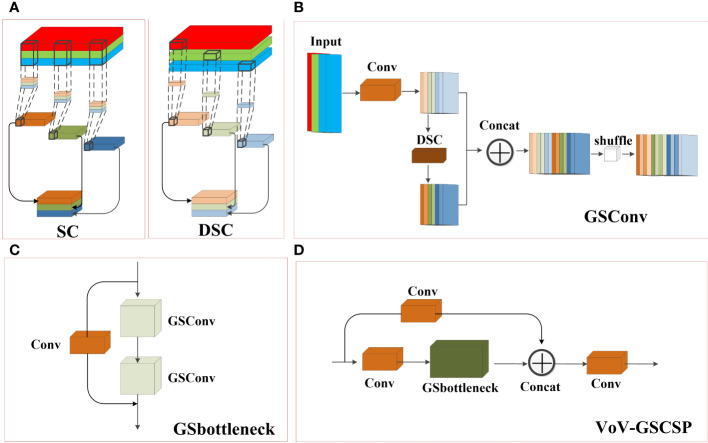
Structural Diagram of **(A)** Standard Convolution (SC) and Depth Separated Convolution (DSC), **(B)** Group Shuffle Convolution(GSConv), **(C)** Group Shuffle bottleneck (GSbottleneck) and **(D)** cross-stage partial network (VoV_GSCSP).

To improve the detection efficiency of the model and reduce the number of model parameters, this study introduces a lightweight convolution method, Group Shuffle Convolution (GSConv) (Li et al., 2022), which can decrease the model complexity while maintaining essentially unchanged accuracy. Current lightweight models typically reduce the number of parameters and FLOPs through Depth Separated Convolution (DSC). However, the channel information of DSC input images is separated during the calculation process. When used DSC alone, it can reduce the feature extraction and fusion capabilities. The convolutional structure of DSC and Standard Convolution (SC) is shown in **Figure 4A**. GSConv is a convolutional method that combines SC and DSC through shuffle, which can mix the information generated by SC into DSC, overcoming the problem of lower feature extraction and fusion capability compared to SC when using DSC alone.

In addition, based on the study of enhancing network learning capabilities such as DensNet (Huang et al., 2017), VoVNet (Lee et al., 2019), and CSPNet (Wang et al., 2020), GSConv was introduced into the bottleneck to form the Group Shuffle bottleneck (GSbottleneck) module. Finally, a one-time aggregation method was used to apply GSbottleneck to the C3 module, forming a cross-stage partial network VoV-GSCSP, which achieved a reduction in model computation without affecting accuracy. When GSConv is applied in a backbone network, it will increase the depth of the network, increasing the computational complexity of each layer, and thus increasing the computational complexity of the model. After passing through convolutional and pooling layers in the feature map of the neck section, the width and height of the feature map are reduced, while the number of channels increases. When the feature map is transmitted through multiple layers of the network, the feature map of the neck section becomes slender, which can be better transformed into more expressive features. Therefore, this study only applies GSConv to the neck section. **Figures 4B–D** illustrates the structural diagrams of GSConv, GSbottleneck, and VoV_GSCSP, respectively.

### 2.5 SPP and SPPF

The Spatial Pyramid Pooling (SPP) layer is instrumental in capturing multi-scale features of the target by focusing on spatial information. Typically integrated into the last layer of a convolutional neural network, the SPP layer divides the input feature map into grids of varying sizes, extracting feature vectors from each grid. This process involves three parallel max-pooling operations and an input branch, followed by concatenating the resulting features to obtain multi-scale representations, which helps the network to better capture the feature information of the target at different scales, and improves the accuracy and robustness of the network.

**Figure 5:**
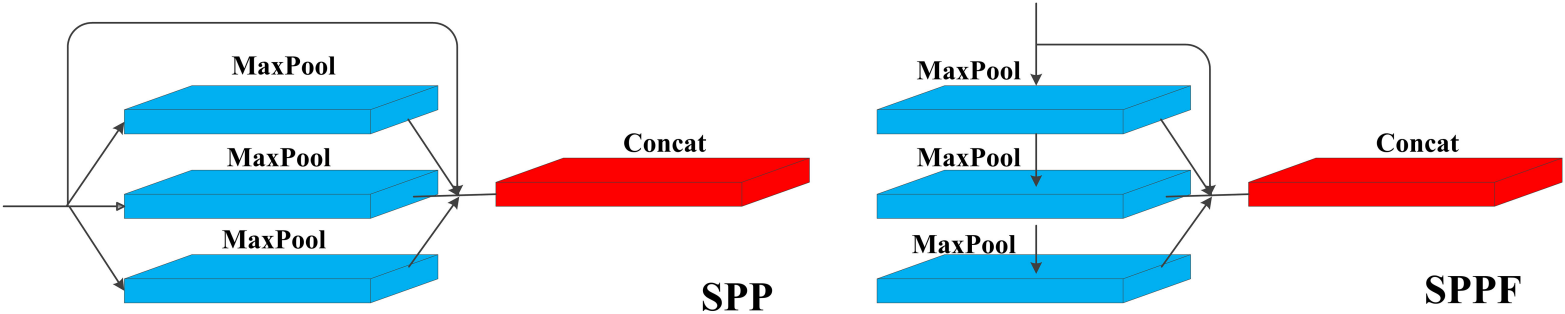
Structure of SPP and SPPF.

SPPF is an optimization of the structure based on SPP, which introduces more pooling layers to improve the performance of the model while including the feature extraction and fusion techniques of the SPP module. Notably, SPPF replaces the parallel branches of SPP with serial connections, sequentially outputting feature vectors layer by layer. This modification includes adding downward output branches to the first and second layers of the three maximally pooled serial connections, thereby increasing the module’s pooling depth. The structure of SPP and SPPF is shown in **Figure 5**. In this study, SPPF is mainly used to solve the problem of too large a scale of object scale change for local and global feature inputs of tea buds. Moreover, SPPF achieves a better balance between performance and speed, which is very suitable for models such as tea buds that need detection accuracy and need to satisfy detection speed. Therefore, this study replaces the SPP in the backbone network with SPPF to enhance the multi-scale feature extraction of the network. Meanwhile, SPPF is applied to the header to deeply extract the deep semantic information introduced by the enhanced feature extraction network, which overcomes the problem that a large amount of low-level semantic information in the shallow network cannot be better fused with the deep semantic information in CA_block.

### 2.6 Maximized partial intersection over union

**Figure 6:**
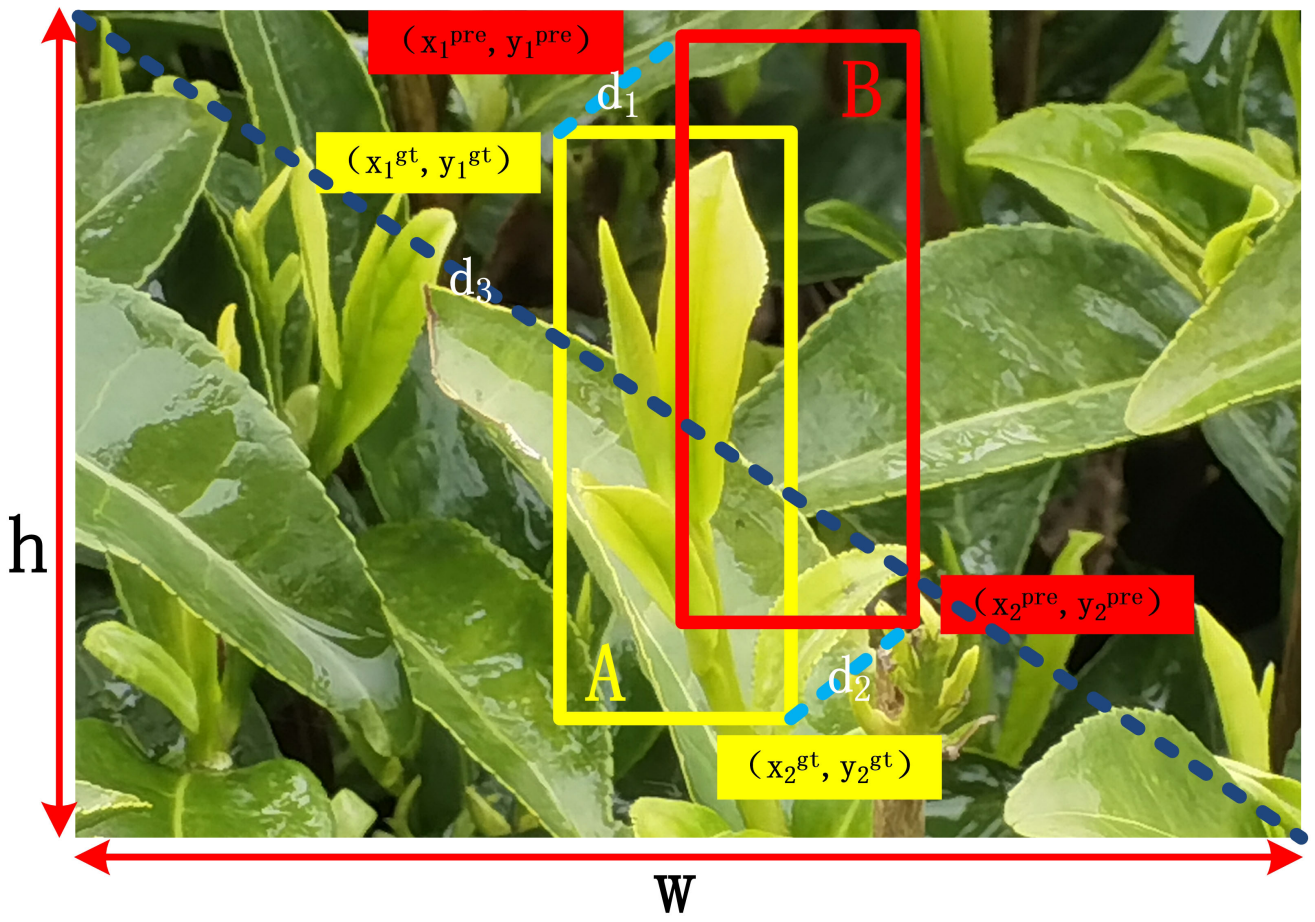
Calculation loss factors for MPDIoU.


To better locate tea buds, this study introduces a new metric, the High Precision Boundary Regression Box (BBR) loss function MPDIoU (Siliang and Yong, 2023), which uses the minimum point distance intersection ratio to calculate the similarity measure between the predicted box and the real box, as shown in **Figure 6**. MPDIoU is an optimization based on Intersection over Union (IoU), which optimizes the calculation method of the overlapping area between the predicted box and the ground truth. It is used to solve the problem of GIoU failure in the initial network when the predicted box and the ground truth overlap highly. IoU is used to calculate the ratio of the intersection and union of predicted boxes and ground truth boxes, and the calculation formula can be described as **Equation 7**.

(7) $$IoU=\frac{A\cap B}{A\cup B}$$

A represents the ground truth box, B represents the predicted box, A∩B represents the area of the intersection area, and A∪B represents the area of the union area.

After obtaining the upper left corner coordinates (x_1_^prd^,y_1_^prd^), lower right corner coordinates (x_2_^prd^,y_2_^prd^) of the prediction box, the upper left corner coordinates (x_1_^gt^,y_1_^gt^), lower right corner coordinates (x_2_^gt^,y_2_^gt^) of the real box, and the width (w) and height (h) of the corresponding feature map, the MPDIoU is calculated as in **Equations 8**–**11**.


(8) $$MPDIoU=IoU-\frac{d_{1}^{2}}{d_{3}^{2}}-\frac{d_{1}^{2}}{d_{3}^{2}}\;$$

(9) $$d_{1}^{2}={\left(x_{1}^{prd}-x_{1}^{gt}\right)}^{2}+{\left(y_{1}^{prd}-y_{1}^{gt}\right)}^{2}$$

(10) $$d_{2}^{2}={\left(x_{2}^{prd}-x_{2}^{gt}\right)}^{2}+{\left(y_{2}^{prd}-y_{2}^{gt}\right)}^{2}$$

(11) $$d_{3}^{2}=w^{2}+h^{2}$$

Among them, d_1_^2^, d_2_^2^, and d_3_^2^ represent the square of the distance between the upper left corner coordinates of the prediction box and the ground truth box, the square of the distance between the lower right corner coordinates of the prediction box and the ground truth box, and the square of the width (w) and height (h) of the corresponding feature map, respectively. The introduction of d_1_^2^, d_2_^2^, and d_3_^2^ is aimed at amplifying the differences between the bounding boxes on both sides, in order to better reflect the positional differences between the two boxes when calculating the similarity between the ground truth box and the predicted box.

Finally, the loss function can be expressed as in **Equation 12**.


(12) Loss=1−MPDIoU

### 2.7 Training environment

The deep learning framework used in this study is PyTorch. The experiments were conducted on a Windows 10 machine with an Intel (R) Core (TM) i7-10700 CPU with a clock speed of 2.90 GHz, 32.0 GB of RAM, and an NVIDIA GeForce RTX3090 24 G GPU. The hyperparameters for model training are shown in **Table 2**.

**Table 2:** Training model hyperparameters.

Method	Batch size	Learning rate	Epochs	Optimizer	Momentum	Weight dedcay
Faster-RCNN	10	1e-3	200	Adam	0.9	0.0005
SSD	10	1e-3	200	Adam	0.9	0.0005
YOLOv3	10	1e-3	200	Adam	0.9	0.0005
YOLOv4	10	1e-3	200	Adam	0.9	0.0005
YOLOv5_m	10	1e-3	200	Adam	0.9	0.0005
Ours	10	1e-3	200	Adam	0.9	0.0005

## 3 Results and Discussion

### 3.1 Evaluating indicator

The detection model in this study was evaluated using precision (P), recall rate (R), and mean average precision (mAP), where P represents the proportion of accurate predictions in all predicted examples and R represents the proportion of accurate predictions in all true examples. The mAP denotes the comprehensive accuracy indicator to evaluate detection model. The formulae are calculated for P, R and mAP as in **Equations 13**–**15**.


(13) $$P=\frac{TP}{\left(TP+FP\right)}$$

(14) $$R=\frac{TP}{\left(TP+FN\right)}$$

(15) $$mAP=\frac{\sum_{1}^{N}\int_{0}^{1}P\left(R\right)dR}{N}$$

TP: Number of positive samples predicted as positive samples

FP: Number of negative samples predicted as positive samples

FN: Number of positive samples predicted as negative samples

N: Indicates the number of types of buds detected (only one type of tea buds is studied in this paper, so N equals 1)

### 3.2 Test comparison before and after model improvement

**Figure 7:**
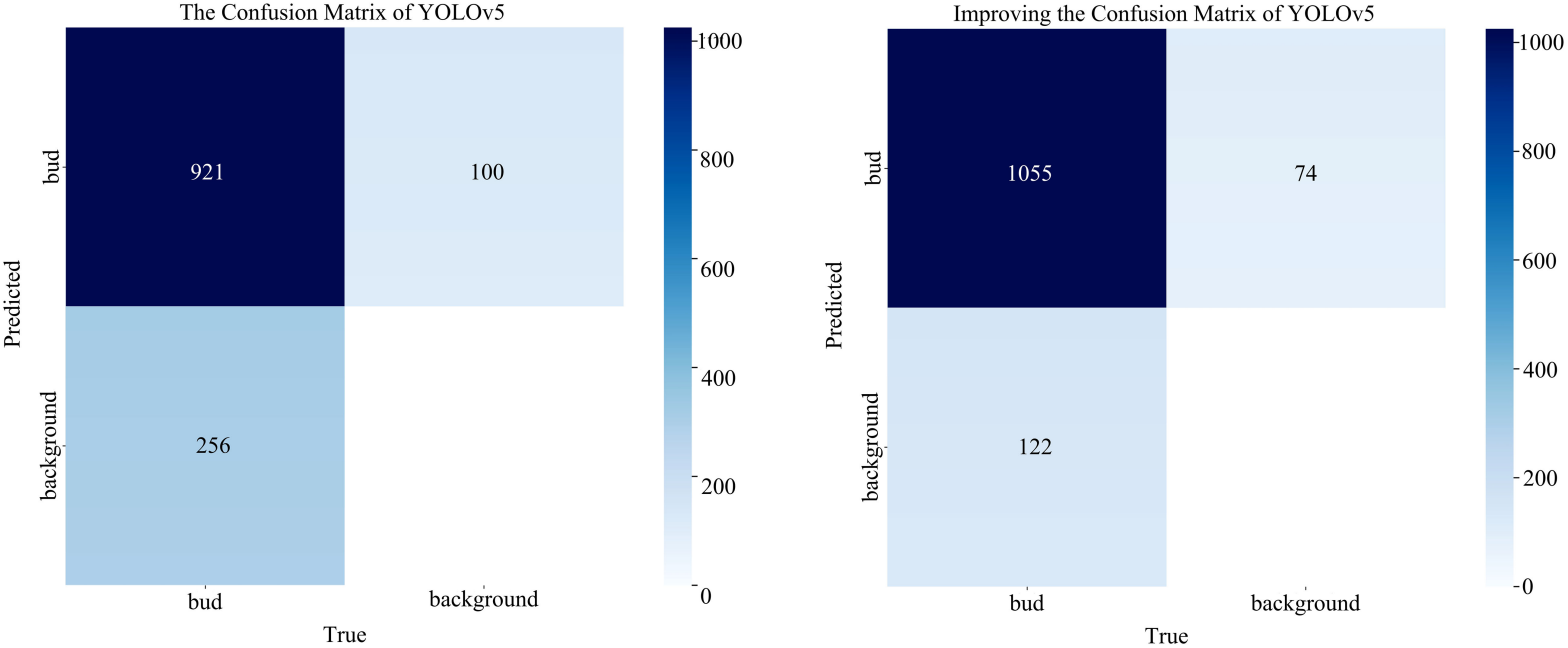
The confusion matrix of the model before and after improvement.

In this paper, the benchmark and improved networks are compared based on the data augmentation set. The test results are presented in **Table 3**. The results indicate that, compared to the initial network, the improved network yielded an increase of 3.26%, 11.43%, and 7.68% for P, R, and mAP, respectively. And the number of parameters decreased by 0.319 M, GFLOPs decreased by 1.343 G, and model size decreased by 1.21 MB. Therefore, it is evident from the comparison before and after model improvement that the improved model can achieve higher accuracy while reducing the complexity of model calculations. Meanwhile, we utilize the confusion matrix to assess the model’s prediction accuracy for tea buds and backgrounds, facilitating a comprehensive evaluation of its performance. **Figure 7** shows the number of correct and incorrect predictions of tea buds on the test set by the model before and after the improvement. The results reveal that the enhanced model increases the number of correctly predicted tea buds by 123 and reduces the misclassification of backgrounds as tea buds by 26, thereby effectively demonstrating the improved detection performance of the model.

**Table 3:** Training results of different target detection methods.

Model	P(%)	R(%)	mAP@0.5(%)	Params(M)	FLOPs(G)	Model size(MB)
Faster-RCNN	56.02	88.95	81.73	136.689	369.719	521.43
SSD	82.80	45.79	69.44	23.612	60.756	90.07
YOLOv3	85.82	66.36	82.28	61.524	65.597	234.69
YOLOv4	87.67	61.00	81.21	63.938	59.953	243.90
YOLOv5_m	90.12	78.25	88.05	21.056	50.598	80.32
**Ours**	**93.38**	**89.68**	**95.73**	**20.737**	**49.255**	**79.11**

Additionally, to visually depict the performance disparity before and after model improvement, this study conducted an analysis of P, R, and mAP using the variance chi-square test. In the ANOVA chi-square test, setting the significance level at 0.05, a probability value lower than this threshold signifies a notable difference in model performance before and after improvement. The probability values of P, R, and mAP before and after model enhancement in this study are 0.000567, 0.002694, and 0.000264, respectively. These values are all below 0.05, indicating a significant difference in model performance before and after the improvement, further confirming that the enhanced model achieves higher detection accuracy.

### 3.3 Grad-CAM visualization

**Figure 8:**
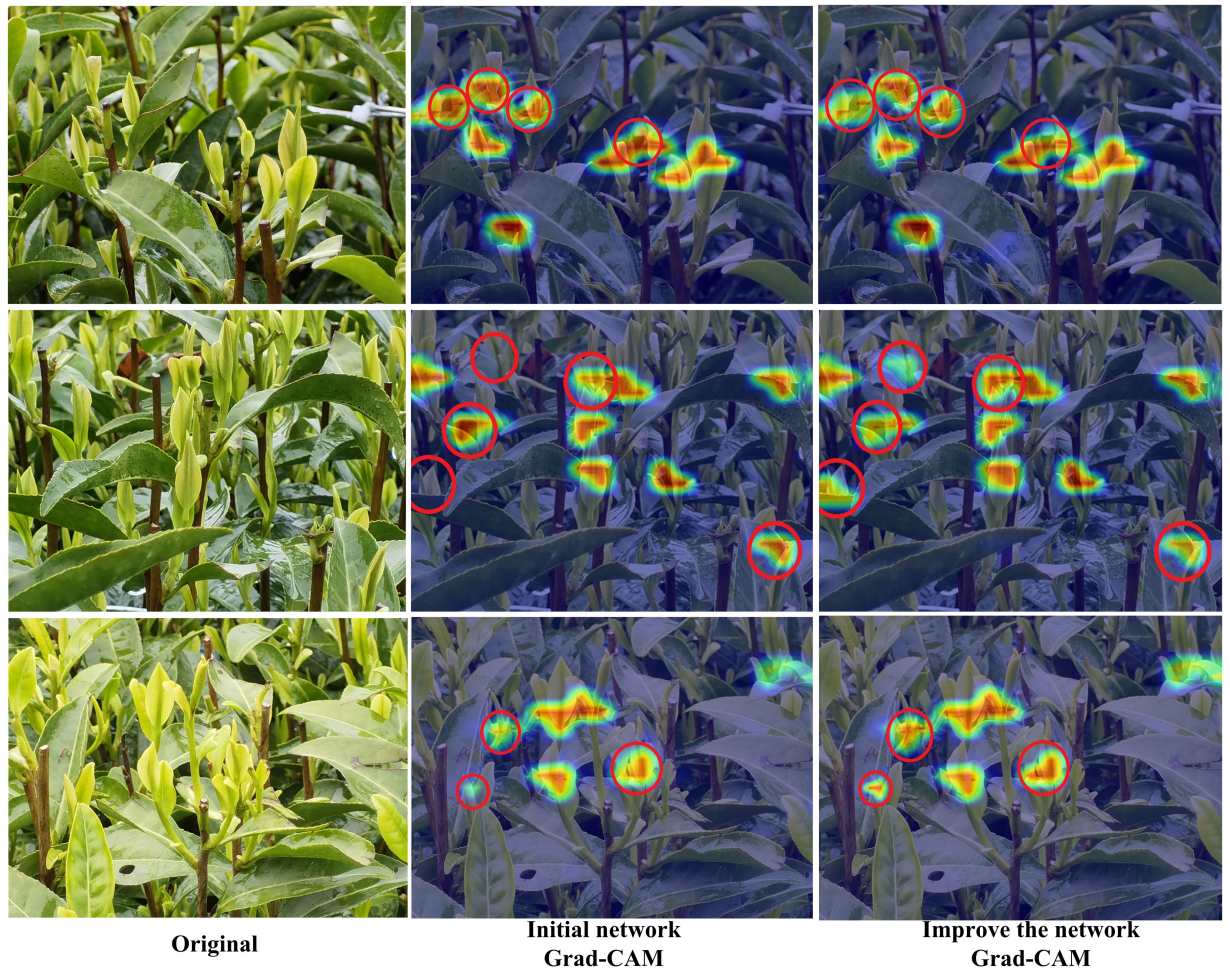
Initial network and improve the network Grad-CAM visualization.

The Grad-CAM (Selvaraju et al., 2017) thermal diagram is used to visualize the regions of interest in the model related to the target when extracting the target features. By displaying the regions of interest through red regions, Grad-CAM can intuitively extract the regions of interest on the tea bud image. In this study, Grad-CAM is used to detect the degree of attention paid to tea bud features before and after model improvement. As the red circle shown in **Figure 8**, by improving the contraction and expansion of the Grad-CAM red regions of the network, the model’s attention is more focused on the tea buds. This weakens the initial network’s focus on background information, and the improved network also learns the characteristics of the buds that the initial network cannot pay attention to, thus proving that the improved network is more effective in paying attention to tea buds. This also verifies that the improved model improves the precision of the network.

### 3.4 Ablation test

The results of the ablation test are presented in **Table 4**, and the P, R, and mAP of the improved network are significantly improved, while the size of the model is also reduced. Through analysis of the data, the result showed that incorporating the CA_block, SPPF, and VoV_GSCSP module into the network significantly improves the P, R, and mAP of the detection network while reducing the model size. This improvement can be attributed to the integration of depth feature information from the VoV_GSCSP module, CA_block, and SPPF module. This integration fully leverages the overall attention of the network to tea buds, improving the detection accuracy of tea buds. Furthermore, the lightweight convolution method of GSConv in the VoV_GSCSP effectively reduces the size of the model.

**Table 4:** Results of the ablation test.

Model	Data enhancement	CA	SPPF	VoV_GSCSP	MPDIoU	P(%)	R(%)	mAP(%)	Model size (MB)
YOLOv5_m						85.20	78.95	87.78	80.32
YOLOv5_m	√					90.12	78.25	88.05	80.32
YOLOv5_m	√	√				91.67	79.52	89.69	80.88
YOLOv5_m	√	√	√			90.89	79.69	89.74	88.28
YOLOv5_m	√	√	√	√		**95.52**	86.92	92.86	79.11
YOLOv5_m	√	√	√	√	√	93.38	**89.68**	**95.73**	**79.11**

The meaning of the symbol "√" is to add the corresponding module to the model.

**Figure 9:**
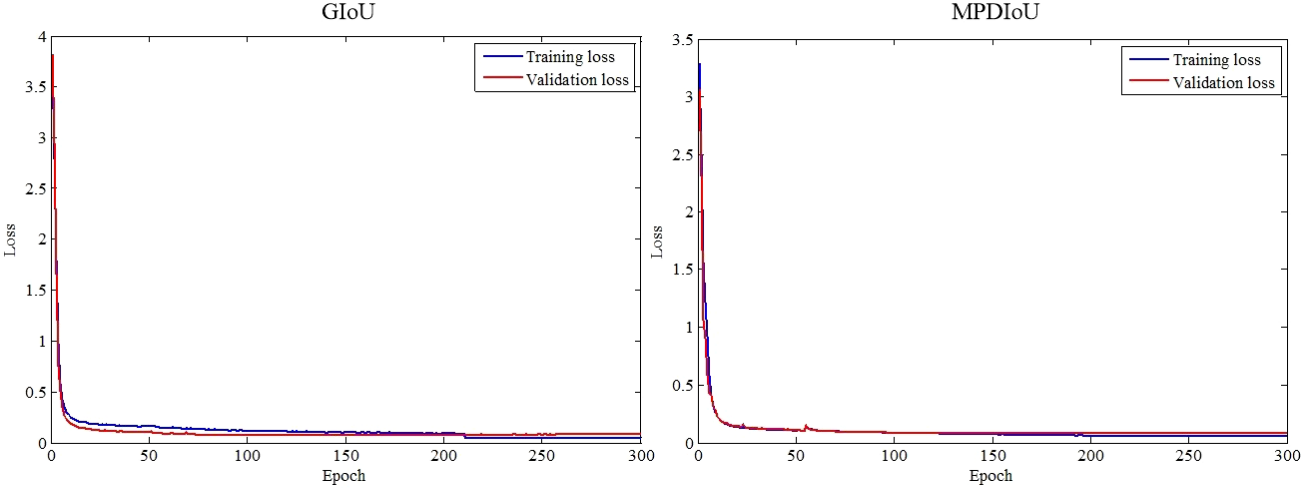
Training and validation loss functions.

From **Figure 9**, a notable contrast emerges between the training loss and validation loss of GIoU, suggesting inadequate learning of tea bud characteristics and resulting in suboptimal performance on the validation set. Conversely, the disparity between the training loss and validation loss of MPDIoU is comparatively minimal, indicating effective learning of tea bud features and yielding satisfactory detection outcomes. Notably, GIoU achieves convergence after approximately 210 epochs of training, while MPDIoU reaches convergence around 190 epochs, showcasing the efficacy of MPDIoU in accelerating model convergence and reducing training duration.

### 3.5 Comparison of different models

This study compares the improved YOLOv5_m model with YOLOv3, YOLOv4, SSD, and Faster R-CNN under data-enhanced conditions. The test results are presented in **Table 3**.

**Figure 10:**
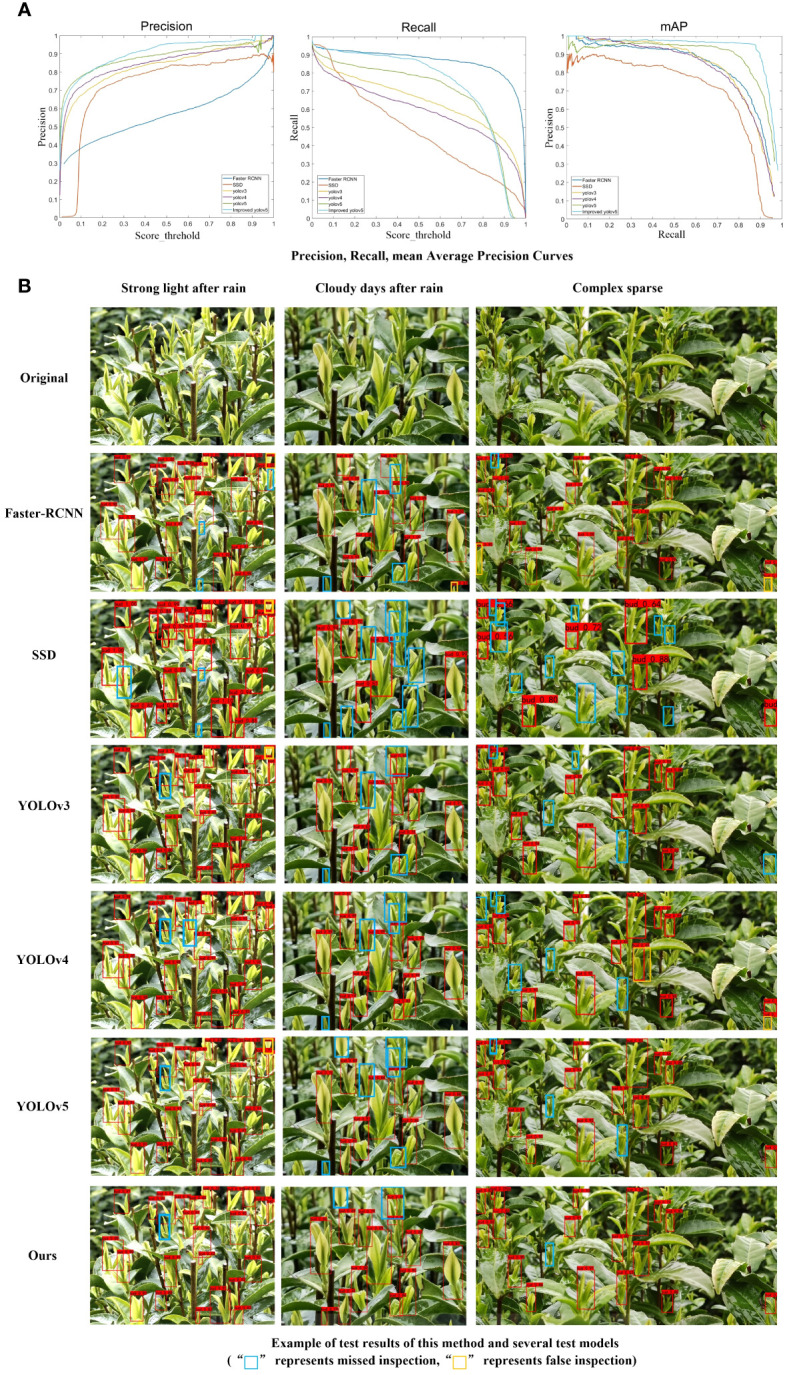
We will replace this sentence with: Detection result of **(A)** Precision, Recall, Average Precision Curves and **(B)** example of test results of this method and several test models.

The results demonstrated that the method proposed in this study outperformed other models in terms of P and mAP. **Figure 10A** illustrates the detection results of P, R, and mAP for all models. Additionally, the proposed method exhibits lower computational complexity in terms of floating-point operations, number of parameters, and model size. Faster R-CNN, as a two-stage detector, tends to produce a high number of false detections when recognizing small targets like tea buds, leading to lower accuracy. Conversely, the SSD model may experience many missed detections during the detection process, resulting in a lower R-value. Although the YOLO series also encounters some missed detections, its performance is comparatively better than SSD and Faster R-CNN. YOLOv5, which is an improvement of YOLOv3 and YOLOv4, is particularly well-suited for detecting small targets, exhibiting higher detection accuracy and faster speed compared to YOLOv3 and YOLOv4. **Figure 10B** displays the detection results of Faster R-CNN, SSD, YOLOv3, YOLOv4, YOLOv5, and the proposed method under varying environmental conditions.

**Figure 10B** shows that the tea buds are densely packed and numerous in a single picture, which increases the difficulty of detection. The method proposed in this study has a better detection effect than other models in terms of precision detection. Although there may still be some omissions, the effect is superior to that of other models, making it suitable for tea bud detection in real tea plantations.

## 4 Conclusion

In this study, due to the density of the tea buds and the complexity of the background environment, existing detection methods struggle to obtain accurate results. To address this issue, we propose an improved YOLOv5_m tea bud detection method to enhance the accuracy and robustness of the detection algorithm. The optimization methods and detection results of this study are as follows:

(1) The fusion of deep feature information from VoV_GSCSP, CA_block, and SPPF modules enhanced the overall attention of the network to tea buds and improved the detection accuracy of tea buds.
(2) Introducing MPDIoU instead of GIoU has achieved rapid convergence of the model and reduced the training time of the model.
(3) The improved YOLOv5_m model achieved a P of 93.38%, a R of 89.68%, and an mAP of 95.73% while maintaining its size or slightly reducing it. These results demonstrate the model’s effectiveness in detecting tea buds. Additionally, the model parameters consist of only 20.737 M, the floating point number is 49.255 G, and the size of the model is 79.11 MB, all of which are superior to other deep learning methods.

The experimental results show that the improved YOLOv5 model has excellent detection performance, which provides technical and theoretical support for automatic picking of high-quality tea leaves. However, the detection accuracy of the model is still a major challenge in complex lighting environments, color interference and old leaf occlusion. Future research will be devoted to collecting tea bud datasets under different scenarios, optimizing the model structure, and improving the ability to detect tea buds under typical unstructured environments in tea gardens. In addition, this study is limited to single-target detection and does not address tea bud categorization (such as single bud, one-leaf-one-bud, or two-leaf-one-bud) or differentiation between different tea varieties. Future work will focus on classifying tea bud classes based on improving the accuracy of tea bud detection, thus enhancing the practical applicability of the model.

## Data availability statement

The raw data supporting the conclusions of this article will be made available by the authors, without undue reservation.

## Author contributions

MJW: Writing – original draft, Writing – review & editing. YL: Conceptualization, Funding acquisition, Methodology, Project administration, Resources, Writing – review & editing. HM: Data curation, Methodology, Writing – review & editing. ZC: Data curation, Methodology, Software, Writing – review & editing. ZG: Conceptualization, Methodology, Software, Writing – review & editing. CD: Writing – review & editing. YPL: Writing – review & editing.
